# Costs of Specialist Referrals From Employer-Sponsored Integrated Health Care Clinics Are Lower Than Those From Community Providers

**DOI:** 10.1007/s11606-022-07724-w

**Published:** 2022-07-26

**Authors:** John R. Wright, Divya K. Madhusudhan, David C. Lawrence, Sharon A. Watts, Daniel J. Lord, Christopher Whaley, Dena M. Bravata

**Affiliations:** 1 Crossover Health, San Clemente, CA USA; 2 Harvard Medical School Postgraduate Medical Education, Global Clinical Scholars Research Training Program, Boston, MA USA; 3 Watts Writing LLC, Akron, OH USA; 4 RAND, Berkeley, CA USA; 5 Stanford Center for Primary Care & Outcomes Research, Palo Alto, CA USA; 6 San Mateo, USA

**Keywords:** referral management, team-based care, health economics, specialty care

## Abstract

**Background:**

There have been very few published studies of referral management among commercially insured populations and none on referral management from employer-sponsored health centers.

**Objective:**

Describe the referral management system of an integrated employer-sponsored health care system and compare specialist referral rates and costs of specialist visits between those initiated from employer-sponsored health clinics and those initiated from community providers.

**Design:**

Retrospective, comparative cohort study using multivariate analysis of medical claims comparing care initiated in employer-sponsored health clinics with propensity-matched controls having specialist referrals initiated by community providers.

**Patients:**

Adult patients (≥ 18 years) eligible for employer-sponsored clinical services incurring medical claims for specialist referrals between 12/1/2018 and 12/31/2020. The study cohort was comprised of 3129 receiving more than 75% of their care in the employer-sponsored clinic matched to a cohort of 3129 patients receiving care in the community.

**Intervention:**

Specialist referral management program implemented by Crossover Health employer-sponsored clinics.

**Main Measures:**

Rates and costs of specialist referrals.

**Key Results:**

The relative rate of specialist referrals was 22% lower among patients receiving care in employers-sponsored health clinics (35.1%) than among patients receiving care in the community (45%, *p* <0.001). The total per-user per-month cost for patients in the study cohort was $372 (SD $894), compared to $401 (SD $947) for the community cohort, a difference of $29 (*p*<0.001) and a relative reduction of 7.2%. The lower costs can be attributed, in part, to lower specialist care costs ($63 (SD $140) vs $76 (SD $213) (*p*<0.001).

**Conclusions:**

Employer-sponsored health clinics can provide effective integrated care and may be able to reduce avoidable specialist utilization. Standardized referral management and care navigation may drive lower specialist spend, when referrals are needed.

**Supplementary Information:**

The online version contains supplementary material available at 10.1007/s11606-022-07724-w.

## BACKGROUND

Primary care providers, especially those hoping to reduce total costs of care for the populations they serve, face several key challenges including acquiring patients and developing longitudinal relationships with them, coordinating care, and managing high-quality referrals.^1–3^ Among these, referral management is among the most important because the costs associated with referrals out of primary care.^4, 5^ Whereas primary care costs account for approximately 5% of total medical expenditures, specialist visits account for 25%.^6^ One-third of primary care visits in the USA result in a specialty referral and such referrals have nearly doubled in the past decade.^7, 8^ Despite the frequency of referrals out of primary care,^9, 10^ more than 25% of patients claim that they do not receive enough information prior to visiting a specialist or do not receive any follow-up after the appointment.^11^

Self-insured employers are embracing the promise of primary care including onsite and near-site clinics as a means of increasing access for their employees to high-quality, cost-effective care.^12^ Patients receive high-quality care when the referral process ensures seamless communication among patients and providers, and when there is a system in place that organizes the multiple steps involved in health care delivery across providers.^9^ Yet, patients, primary care physicians, and specialists alike report that referral management often falls short in this regard.^4^ Referral management from primary care has been a longstanding challenge because it requires access to comprehensive provider networks, cost, and quality information.^5, 10^ Even among commercial accountable care organizations, the failure to consistently demonstrate cost savings has been associated with barriers to improving referral patterns.^13^ Unfortunately, there have been very few published studies of referral management among commercially insured populations and none on referral management from employer-sponsored health centers.^14–16^

The purpose of this paper is to first describe the referral management process by a national provider of employer-sponsored health centers offering in-person and virtual primary care, physical medicine, behavioral health, and health coaching. We then compare the rates and costs of specialty referrals for commercially insured patients receiving their primary care in employer-sponsored clinics versus in the community. This study can inform the development of referral management processes for in-person and virtual primary care for employee populations.

## METHODS

### Referral Management Intervention

We studied the referral management program implemented by Crossover Health for adult (age >18 years) patients with employer-sponsored health insurance seen in their clinics between December 1, 2018, and December 31, 2020. The referral management system was implemented gradually across Crossover Health clinics in June 2017 and was in place in all clinics by June 2018. Crossover Health provides integrated primary care, physical medicine, behavioral health care, and health coaching through employer-sponsored onsite and near-site clinics.^17, 18^ Patients may self-refer into any internal specialty, they may be referred between internal specialties (e.g., from a primary care provider to a psychologist), or they may be referred to specialists outside the employer-sponsored clinical service offering.

Historically, an ordering clinician specified the exact provider to whom the patient was being referred. Serving a geographically distributed population in this manner requires knowledge of high-quality, in-network providers across a range of specialties and geographies which is impossible for any individual clinician. Instead, centralizing this function with a team of dedicated care navigators can standardize processes and increase referral efficiency. All referrals to providers including laboratories, imaging centers, and specialists outside the employer-sponsored clinic were managed by care navigators. Clinicians had no incentives to either limit or promote internal or external referrals.

Care navigators included individuals with a range of healthcare experiences (e.g., some with no prior medical experience, medical assistants, nurses). They all received a standardized 12-week training in steering patients to high-quality, cost-effective options (e.g., in-network rather than out-of-network providers, outpatient imaging and endoscopy centers rather than hospital-based alternatives). Care coordinators were responsible for communicating with the referring members of the care team, the patient, and the specialist. They developed regional expertise about providers, were facile in the use of navigation tools to identify high-quality providers outside the geographic region of a physical location (such as those made available by employers as part of the health benefits for their employees), identified local resources to address care barriers related to social determinants of health, and helped patients navigate their employer benefits.

The referral directory used by navigators in the employer-sponsored clinics was a continuously improved list of specialists and facilities. Navigators used standard criteria to curate this list which included updating information about provider quality (e.g., reviewing board-certifications, malpractice or licensure issues), confirming the ability of the provider to see patients in a timely manner, verifying in-network status, and documenting key information from conversations with office staff (e.g., recent changes in office practices due to COVID-19) and online ratings/reviews. They also aggregated ratings from internal patient satisfaction and quality surveys from referring providers after every referral.

Navigators managed the transfer of records among providers (e.g., providing clinical data from the requesting provider to specialists, obtaining specialists’ recommendations and notes for the requesting provider) and “closed the referral loop” by ensuring that the referral occurred or if not, documenting the reason why not. Navigators leveraged relationships with preferred specialty clinics to streamline the scheduling process for urgent referrals (to be seen within 72 h) and non-urgent (to be seen within 7 days).

### Data and Statistical Methods

The key outcomes of interest of this analysis were the rates of specialist referrals and cost of those referrals in the employer-sponsored clinic and community cohorts. We used multiple sources of patient data for this analysis, including insurance eligibility files, medical claims, and the electronic health record. All Crossover clinics operate on a single electronic health record platform; we did not receive electronic health record information for patients seen in the community. Medical claims and eligibility files from different sources and/or payors were combined in a single data platform.

We defined specialist services as professional visits other than primary care, behavioral health, physical medicine, or health coaching. To estimate the specialist referral rate, we calculated the proportion of specialist visits of all professional visits for the employer-sponsored clinic cohort compared to the community cohort.

The amount that patients pay for care from the employer-sponsored clinics depends on the benefits they receive through their employer; however, the majority pay a copay or coinsurance amount that is similar to or less expensive than community providers ranging between $0 and $90 per visit. Costs for specialist referrals were calculated based on allowed amount on a patients’ claims. The employer-sponsored clinic receives the same compensation for in-person and virtual visits. For purposes of this study, total cost of care was defined as total allowed claims plus an allocation of the revenue received by the employer-sponsored clinic. Revenue allocations are calculated as the total revenue divided by total visits in the measurement period, multiplied by each study member’s total visits to the clinic.

We measured patient satisfaction for the care received in the employer-sponsored clinics with a 4-item self-reported patient satisfaction survey rated on a 5-point Likert scale (possible score: 0 to 100% satisfied) and also asked a single item Net Promoter Score (NPS) question, “How likely is it that you would recommend the health center to a friend or colleague?^19^ Further, we measured patient satisfaction for the specialist referrals using ten questions (Appendix Table 1) rated on a 5-point Likert scale. We surveyed patients about their wait times at the specialist office but did not assess NPS for specialist visits. We did not survey patients who received care in the community.

### Cohort Definitions

This study used two separate matched samples to examine patient- and encounter-level outcomes. We used the first cohort analysis to examine overall specialist utilization and total cost of care trends. We identified 3129 patients who received most of their primary care services (defined as greater than 75%) from Crossover providers during the study interval. Patients who were eligible for services during the study interval but did not access primary care through Crossover were considered community controls (*n*= 52,393) for a propensity-matched study. Propensity scores were generated with logistic regression of attribution to the study cohort conditional on gender, age, employer, patient type (employee or spouse/dependent), risk score, commute distance from the nearest employer-sponsored clinic site and Department of Health and Human Services-Hierarchical Condition Categories (HHS-HCC) chronic conditions.^20^ Matching was performed using nearest neighbors.^20^ In this analysis, we compared the total costs for the 3129 patients in the Crossover cohort and 3129 matched patients in the community cohort. Notably, these are not paired matches (i.e., there are no direct comparisons between individuals in the cohorts) as all comparisons were at the cohort level.

We used the second cohort analysis to examine referral-related outcomes. We analyzed encounters from Crossover’s referral management program and propensity-matched them to encounters that were not initiated by the referral management program. Referrals were restricted to the ten most common referral types. Propensity scores were computed using logistic regression of calendar year, patient employer, gender, age, principal procedure code and principal diagnosis code grouping. Diagnosis code groupings were Care Process Families from Health Catalyst’s Care Process Hierarchy of ICD-10.^21, 22^ We performed a generalized linear regression analysis (gamma model, to address skewness) of propensity-matched specialist encounters conditional on calendar year, employer, gender, age, and HHS-HCC risk scores.

All statistical analyses were conducted in R version 4.0.3 (2020-10-10). Given that all data are routinely collected for ongoing patient care, this protocol was considered IRB exempt.

## RESULTS

### Demographics

Of the 6,258 patients included in the analysis, 47% were male with a mean age of 39.5 years (SD 10.7 years) (Table 1). On average, they lived 11.6 miles (SD 20.5 miles) from the nearest employer-sponsored health clinic, and they were generally healthy (average HHS-HCC risk score 1.7 (1.9 SD)). The community cohort was well balanced on all these characteristics.

**Table 1:** Patient Demographics

Characteristics	Employer-sponsored clinic cohort	Community cohort
*N*	3129	3129
Gender (% male)	46.6%	46.9%
Age (SD) (years)	39.5 (10.7)	39.3 (10.8)
Distance to employer-sponsored clinic from patient’s home (SD) (miles)	11.6 (20.5)	11.2 (15.6)
HHS-HCC risk score (SD)	1.7 (1.9)	1.6 (1.6)

### Referral Rates

#### Internal Referrals

**Figure 1:**
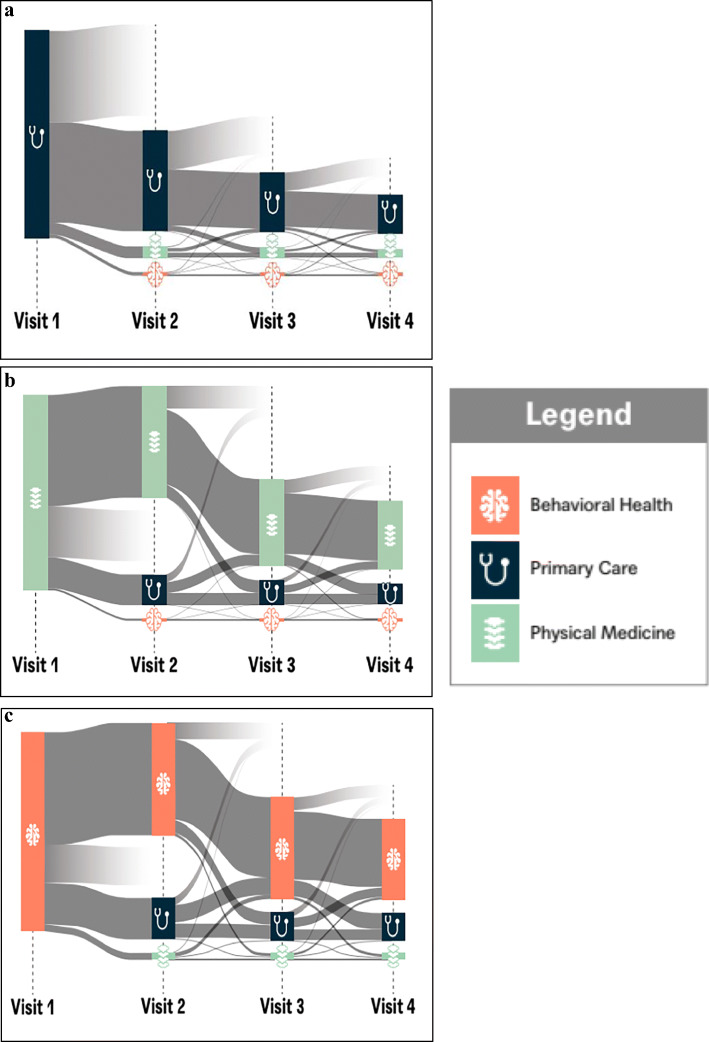
Patient paths among internal referrals. (a) Patient paths from primary care. (b) Patient paths from physical medicine. (c) Patient paths from behavioral health. Legend: These schematics describe the flow of patients from an initial visit with providers in one clinical discipline to the other disciplines at the employer-sponsored clinics. Each patient is denoted by a gray line; thus, the size of the gray bar represents the proportion of patients experiencing primary care at first visit and transitioning to the same/other clinical disciplines for subsequent visits. Lines that end in white denote the end of an episode of care. Because each panel represents all of the referrals originating out of each clinical discipline, it is important to note that they are not on the same scale (since there were vastly more patients seen in primary care than in behavioral health).

Among the patients seen in the provider-sponsored clinics, 5.9% were referred to another internal clinician. The most common internal referral paths were from primary care to physical medicine (29% of all internal referrals, Table 2, Fig. 1a). Interestingly, there were numerous intradepartmental referrals. For example, within physical medicine chiropractors may care for a patient with back pain for acute symptom relief and then refer that patient on to physical therapy for rehabilitation (Fig. 1b). Similarly, primary care providers (PCPs) may refer to other PCPs who specialize in weight loss or sports medicine and within behavioral health, psychotherapists may refer patients to a psychiatrist for medication management. Although relatively few internal referrals were made by behavioral health providers, 22% were for health coaching, 20% were for primary care, and 7% were for physical medicine (Fig. 1c).

**Table 2:** Internal Referrals Within the Employer-Sponsored Health Center

Initial clinician	Internal referral to this specialist				
	Primary care	Physical medicine	Behavioral health	Health coaching	Total of all 1internal referrals
Primary care	2%	29%	10%	13%	54%
Physical medicine	5%	19%	3%	2%	29%
Behavioral health	1%	1%	3%	2%	6%
Health coaching	1%	1%	1%	1%	4%

Legend: Referrals to clinical disciplines other than the core clinical offering at employer-sponsored health centers (such as dermatology, optometry, and nursing) are not included in the table. The reason that this table only represents 93% of all internal referrals is that it does not include the internal referrals to services such as dental care and dermatology which are only offered at selected employer-sponsored clinics.

#### Specialist Referrals

**Figure 2:**
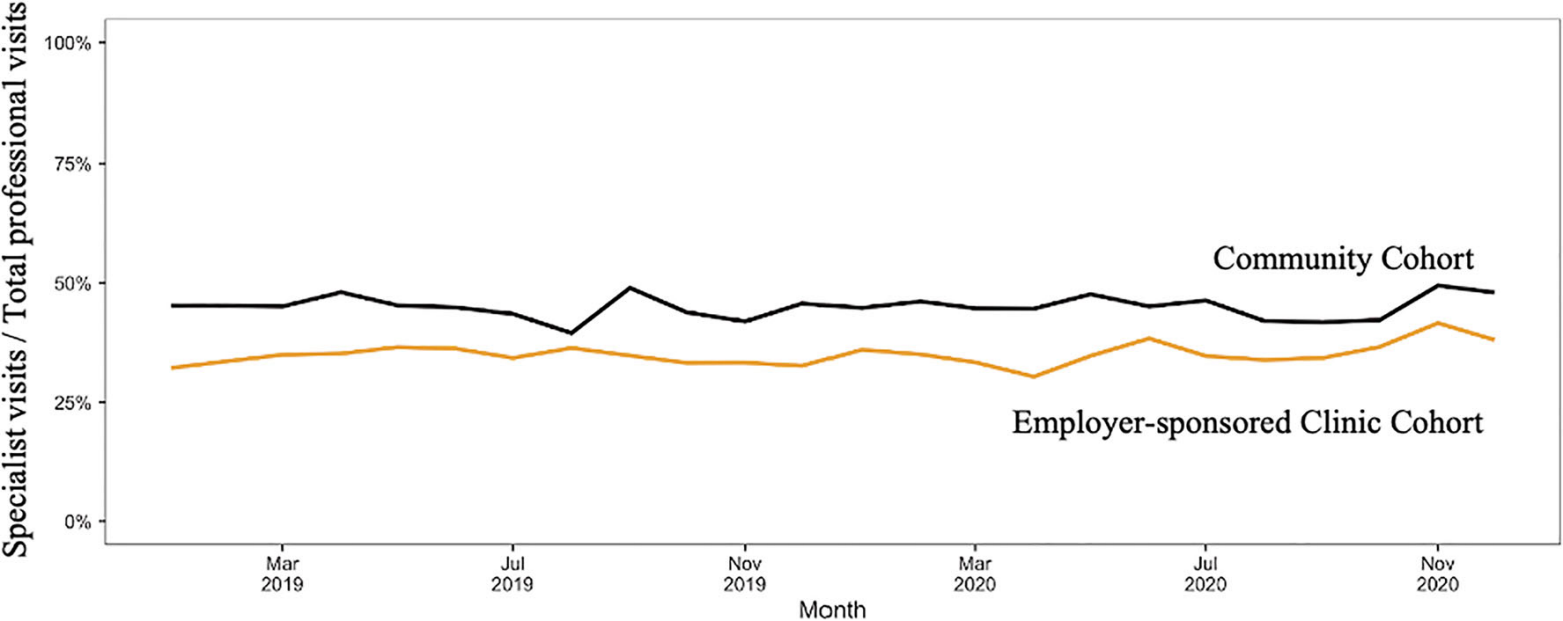
Specialist referral rates over time.

The relative rate of specialist referrals was 22% lower among patients receiving care in employers-sponsored health clinics (35.1%) than among patients receiving care in the community (45%, *p* <0.001) (Fig. 2). The most common referrals were for imaging (36%), obstetrics/gynecology (13%), gastroenterology (11%), and otolaryngology (11%) (Table 3).

**Table 3:** Top Ten Specialist Referrals and Their Costs

Specialist	*N* (%)	Average cost of specialist visit initiated in the employer-sponsored clinic cohort (SD)	Average cost of specialist visit initiated in the community cohort (SD)
Imaging	11544 (36%)	$242 ($418)	$264 ($494)
Obstetrics and gynecology	4228 (13%)	$191 ($209)	$193 ($223)
Otolaryngology	3450 (11%)	$259 ($202)	$274 ($228)
Gastroenterology	3590 (11%)	$272 ($323)	$317 ($499)
Allergy, asthma, and immunology	2340 (7%)	$284 ($216)	$311 ($244)
Cardiology	1966 (6%)	$325($376)	$322 ($388)
Dermatology	1698 (5%)	$265 ($329)	$238 ($247)
Urology	1482 (5%)	$217 ($177)	$226 ($199)
Orthopedics	1166 (4%)	$209 ($198)	$206 ($190)
Sleep medicine	446 (1%)	$344 ($235)	$442 ($549)
**Overall**	**31910 (100%)**	**$249 ($329)**	**$266 ($394)**

#### Costs

The average per user per month (PUPM) total cost of care in the employer-sponsored clinic was $372 (SD $894) compared with $401(SD $947) (*p*<0.001) for employees receiving primary care in the community, a difference of $29 (*p*<0.001) (Table 4) and a relative reduction of 7.2%. The lower costs can be attributed in part to lower specialist care costs ($63 (SD $140) vs $76 (SD $213) (*p*<0.001). On average, specialist visit costs were 3.6% lower (SE 1.5%) when a referral was initiated by care navigators as compared to those initiated in the community (Appendix Table 2).

**Table 4:** Comparison of Costs of Care

Characteristics	Employer-sponsored clinic cohort (SD)	Community cohort (SD)	Regression estimate as %	*p*-value
Total per user per month cost	$372 ($894)	$401 ($947)	−14.1%	0.002
Primary care cost per user per month	$91 ($110)	$69 ($99)	23.8%	<0.001
Specialty care cost per user per month	$63 ($140)	$76 ($213)	−17.9%	<0.001
Hospital, urgent care, and ASC cost per user per month	$142 ($669)	$159 ($587)	−7.4%	0.34**
Pharmacy cost per user per month	$76 ($389)	$97 ($525)	−10%	0.48**
Specialist utilization rate	35.1%	45.0%	−0.1*	<0.001

Legend: Cost of care was modeled from the propensity-matched data. In this GLM-gamma regression, the dependent variable was the encounter cost per month and the independent variables were employer, patient age, patient gender, patient risk score, patient type and commute distance from the nearest employer-sponsored clinic site. For subcategory cost of care regressions, non-users of the employer-sponsored clinics were excluded.

*Specialist utilization rate was modeled in a separate linear regression of the propensity-matched cohorts conditional on month and year fixed effects.

**NS, not significant

#### Wait Times

Most patients (94.8%) receiving care in the employer-sponsored clinics waited less than 5 min at their medical appointment. Most patients requiring specialist care (82.3%) waited less than 10 min at their medical appointment before seeing the referred specialist (Appendix Table 3).

#### Patient Satisfaction

Self-reported patient satisfaction was high with both the care received in the employer-sponsored clinics (93.8%, SD 0.28%; NPS 87.04) and with specialist referrals (4.33 (SD 0.11)) (Appendix Table 1).

## DISCUSSION

This, the first published evaluation of referral management at a national, integrated employer-sponsored care provider, offers three key findings. First, providing an integrated offering of primary care, physical medicine, and behavioral health in employer-sponsored clinic settings may provide easier access to these highly used services and results in high patient satisfaction. Patients often received care by more than one clinician in the employer-sponsored clinics. According to a recent study by the Business Group on Health, a national coalition of self-insured employers, 57% of self-insured employers are considering implementing onsite or near site primary care by 2024.^23^ Given that the most common internal referrals in our analysis were from primary care to physical medicine (29%), employer-sponsored primary care offerings should consider including physical medicine services such as chiropractic and physical therapy.

Second, patients receiving care from integrated employer-sponsored clinic providers have significantly fewer referrals to specialists than those receiving care in the community (35.1% vs 45%), a relative difference of 22%. ​The community referral rate is consistent with referral rates in the USA, where more than a third of patients are referred to a specialist each year.^4^ Although there were no specific restrictions on specialist referrals for employer-sponsored clinic staff, it may be that the overall culture of evidence-based medicine and protocolized care contributed to this lower referral rate.

Third, after accounting for the operational costs of the clinics, the total per-user-per-month costs for patients receiving care through employer-sponsored health clinics were lower than among those receiving care in the community ($372 compared with $401), a relative reduction of 7.2%. While the drivers of these savings are multifactorial, this finding supports previous studies that demonstrate the benefits of implementing care navigation initiatives that connect complex and vulnerable patient populations with the care they need^24, 25^ and have been associated with reduced episode of care costs.^26^ Given the diversity of care navigator models,^24^ however, further research is needed to map specific configurations of care navigator programs onto savings for employers.

The widespread adoption of telehealth for commercially insured populations in response to the COVID-19 pandemic will only increase the need for appropriate referral management. Specifically, referral management for employers with geographically distributed populations requires access to high-quality, in-network providers in all geographies where the virtual care is being delivered. This can be challenging for organizations that historically provided national tele-urgent care (since urgent care typically requires relatively few referrals), as well as those that provided local brick-and-mortar primary care (who may have had only local referral capabilities) who are now expanding the geographic reach of their services virtually. Self-insured employers are embracing the promise of virtual primary care, often under value-based payment models, as a means of increasing access for their employees to high-quality, cost-effective care.^23^ For virtual primary care to fulfill its promise of helping employee populations get the preventive services and chronic condition management they need, virtual primary care offerings must solve the key issue of managing referrals for labs, imaging and other tests, and specialist care. We believe that our results are generalizable to value-based care organizations that adopt a similar structured approach to referral management.

This study had four key limitations. First, the risk model used in this analysis is based on health care claims and may have underestimated the risk of the study cohort given that patients seen in the employer-sponsored clinics do not have comprehensive claims submitted for their care. This underestimated the difference in cost between care for patients in the study and community cohorts. Second, because we did not include physical medicine or behavioral health claims in the comparative analysis, we lacked the ability to specifically evaluate the costs for this care in the community. Given the prevalence of physical medicine and behavioral health care utilization, this should be a key target for future research. Third, because this analysis included visits from December 1, 2018, to December 31, 2020, it included the months when health care utilization was affected by the COVID-19 pandemic. Our matched cohort analysis accounted for this so the comparisons between the two groups would not have been affected; however, the absolute rates of referrals in both cohorts would likely have been lower than during non-pandemic periods. Finally, we did not survey patients receiving care in the community. Thus, we were unable to assess their satisfaction or experience with specialist referrals.

Our research supports the work of others that demonstrates that integrating the most common services in such a way that promotes multidisciplinary collaboration reduces the overall cost of care.^27–29^ Populations cared for in integrated, employer-sponsored health clinics may require fewer specialist visits and when such visits are required, the use of standardized referral management process may result in lower specialist referral costs.

## Supplementary information

ESM 1 (DOCX 25 kb)
