# Supplementary material for: Costs of Specialist Referrals From Employer-Sponsored Integrated Health Care Clinics Are Lower Than Those From Community Providers
Source: J Gen Intern Med. 2022 Jul 26;37(15):3861–8. doi: 10.1007/s11606-022-07724-w (PMC9321287; doi:10.1007/s11606-022-07724-w)
Supplement: Supplementary file 1 — (DOCX 25 kb) [file 11606_2022_7724_MOESM1_ESM.docx]

**Appendix Table 1. Patient Satisfaction with Specialist Referrals**

| **Questionnaire Item** | **Mean** | **SD** | **Min** | **Max** |
| --- | --- | --- | --- | --- |
| Comfortable and inviting environment | 4.3 | 0.88 | 1 | 5 |
| Confident in the care quality | 4.3 | 0.93 | 1 | 5 |
| I felt my privacy was protected | 4.5 | 0.71 | 1 | 5 |
| I left knowing what to do | 4.3 | 0.84 | 1 | 5 |
| My issues were addressed | 4.2 | 0.98 | 1 | 5 |
| Scheduling was simple and convenient | 4.2 | 1.00 | 1 | 5 |
| Specialist was easy to talk to | 4.5 | 0.84 | 1 | 5 |
| The appointment time you were able to get was convenient | 4.3 | 0.80 | 1 | 5 |
| The check-in process was easy and effective | 4.4 | 0.76 | 1 | 5 |

**Appendix Table 2. Regression Analysis of Specialist Visit Costs**

| **Parameter** | **% Estimate** | **Standard Error** | **t-statistic** | **p-value** |
| --- | --- | --- | --- | --- |
| Intercept | 22221.3% | 0.089 | 60.998 | 0.000 |
| Employer | -39.6% | 0.019 | -26.995 | 0.000 |
| Year 2019 | 31.7% | 0.076 | 3.628 | 0.000 |
| Year 2020 | 23.2% | 0.076 | 2.758 | 0.006 |
| Year 2021 | 31.8% | 0.076 | 3.629 | 0.000 |
| Age (Years) | 0.3% | 0.001 | 3.360 | 0.001 |
| Male | 2.3% | 0.016 | 1.403 | 0.161 |
| Risk Score | 1.1% | 0.002 | 4.663 | 0.000 |
| Specialty - Cardiology | 9.5% | 0.042 | 2.132 | 0.033 |
| Specialty - Dermatology | -10.9% | 0.044 | -2.613 | 0.009 |
| Specialty - Gastroenterology | 3.8% | 0.037 | 1.008 | 0.313 |
| Specialty - Imaging | -10.0% | 0.032 | -3.357 | 0.001 |
| Specialty - OBGYN | -33.3% | 0.036 | -11.235 | 0.000 |
| Specialty - Orthopedics | -29.3% | 0.050 | -6.996 | 0.000 |
| Specialty - Otolaryngology | -9.0% | 0.037 | -2.539 | 0.011 |
| Specialty - Sleep Medicine | 28.6% | 0.071 | 3.525 | 0.000 |
| Specialty - Urology | -24.7% | 0.046 | -6.190 | 0.000 |
| **Care Navigation Referrals** | **-3.6%** | **0.015** | **-2.395** | **0.017** |

**Appendix Table 3. Comparison of Wait Times at Specialist Referrals**

| **Wait times** | **Employer sponsored clinics**  **(N= 265)** | **Specialist referrals**  **(N= 1253)** |
| --- | --- | --- |
| 1 to 5 minutes | 34.8% | 36.6% |
| 5 to 10 minutes | 4.4% | 32.5% |
| Did not wait | 60% | 13.2% |
| ﻿ > 10 minutes | 1.1% | 17.7% |

**Appendix Table 4. Prevalence of Conditions in the Cohorts**

| **Condition** | **Crossover patients** | **Matched Cohort** |
| --- | --- | --- |
| Anorexia/Bulimia Nervosa | 0.10% | 0.10% |
| Disorders of the Immune Mechanism | 0.50% | 0.30% |
| Spinal Cord Disorders/Injuries | 0.10% | 0.10% |
| Asthma | 5.80% | 4.60% |
| Breast (Age 50+) and Prostate Cancer, Benign/Uncertain Brain Tumors, and Other Cancers and Tumors | 0.80% | 0.40% |
| Diabetes without Complication | 2.20% | 1.10% |
| Chronic Hepatitis, Except Chronic Viral Hepatitis C | 0.40% | 0.40% |
| Inflammatory Bowel Disease | 0.70% | 0.40% |
| Intestinal Obstruction | 0.30% | 0.10% |
| Congestive Heart Failure | 0.60% | 0.20% |
| Cardio-Respiratory Failure and Shock, Including Respiratory Distress Syndromes | 0.40% | 0.10% |
| Heart Assistive Device/Artificial Heart | 0.00% | 0.00% |
| Pulmonary Embolism and Deep Vein Thrombosis | 0.40% | 0.20% |
| Autistic Disorder | 0.60% | 0.00% |
| Specified Heart Arrhythmias | 0.90% | 0.50% |
| Diabetes with Chronic Complications | 1.90% | 0.70% |
| Rheumatoid Arthritis and Specified Autoimmune Disorders | 0.80% | 0.40% |
| Major Congenital Heart/Circulatory Disorders | 0.30% | 0.20% |
| Major Depressive and Bipolar Disorders | 1.70% | 1.30% |
| Liver Transplant Status/Complications | 0.00% | 0.00% |
| Amyloidosis, Porphyria, and Other Metabolic Disorders | 0.10% | 0.10% |
| Completed Pregnancy With Complications | 1.50% | 1.00% |
| Acquired Hemolytic Anemia, Including Hemolytic Disease of Newborn | 0.10% | 0.10% |
| Thyroid Cancer, Melanoma, Neurofibromatosis, and Other Cancers and Tumors | 0.40% | 0.20% |
| Atrial and Ventricular Septal Defects, Patent Ductus Arteriosus, and Other Congenital Heart/Circulatory Disorders | 0.40% | 0.20% |
| Miscarriage with No or Minor Complications | 0.70% | 0.60% |
| Colorectal, Breast (Age < 50), Kidney, and Other Cancers | 0.30% | 0.10% |
| Seizure Disorders and Convulsions | 0.80% | 0.20% |
| Completed Pregnancy with No or Minor Complications | 2.50% | 2.00% |
| Hypoplastic Left Heart Syndrome and Other Severe Congenital Heart Disorders | 0.00% | 0.00% |
| Sickle Cell Anemia (Hb-SS) | 0.00% | 0.00% |
| Chronic Obstructive Pulmonary Disease, Including Bronchiectasis | 0.40% | 0.10% |
| Drug Dependence | 0.40% | 0.10% |
| Paraplegia | 0.00% | 0.00% |
| Kidney Transplant Status | 0.00% | 0.00% |
| Schizophrenia | 0.10% | 0.00% |
| Coagulation Defects and Other Specified Hematological Disorders | 0.70% | 0.40% |
| Systemic Lupus Erythematosus and Other Autoimmune Disorders | 0.50% | 0.30% |
| Ischemic or Unspecified Stroke | 0.20% | 0.20% |
| Metastatic Cancer | 0.20% | 0.10% |
| Acute Pancreatitis/Other Pancreatic Disorders and Intestinal Malabsorption | 0.90% | 0.40% |
| Chronic Ulcer of Skin, Except Pressure | 0.20% | 0.10% |
| Adrenal, Pituitary, and Other Significant Endocrine Disorders | 0.90% | 0.60% |
| Septicemia, Sepsis, Systemic Inflammatory Response Syndrome/Shock | 0.30% | 0.20% |
| Hemophilia | 0.00% | 0.00% |
| Protein-Calorie Malnutrition | 0.20% | 0.10% |
| Term or Post-Term Singleton Newborn, Normal or High Birthweight | 0.60% | 0.00% |
| HIV/AIDS | 0.20% | 0.10% |
| Combined and Other Severe Immunodeficiencies | 0.00% | 0.00% |
| Myasthenia Gravis/Myoneural Disorders and Guillain-Barre Syndrome/Inflammatory and Toxic Neuropathy | 0.10% | 0.10% |
| Acute Myocardial Infarction | 0.20% | 0.00% |
| Vascular Disease with Complications | 0.10% | 0.00% |
| Peritonitis/Gastrointestinal Perforation/Necrotizing Enterocolitis | 0.10% | 0.10% |
| Opportunistic Infections | 0.10% | 0.00% |
| Congenital/Developmental Skeletal and Connective Tissue Disorders | 0.20% | 0.10% |
| Non-Hodgkin's Lymphomas and Other Cancers and Tumors | 0.20% | 0.10% |
| Cirrhosis of Liver | 0.10% | 0.00% |
| Thalassemia Major | 0.00% | 0.10% |
| Mucopolysaccharidosis | 0.00% | 0.00% |
| Multiple Sclerosis | 0.20% | 0.10% |
| Chronic Kidney Disease, Severe (Stage 4) | 0.00% | 0.00% |
| Hip Fractures and Pathological Vertebral or Humerus Fractures | 0.00% | 0.10% |
| Hydrocephalus | 0.00% | 0.00% |
| Cleft Lip/Cleft Palate | 0.00% | 0.00% |
| Fibrosis of Lung and Other Lung Disorders | 0.20% | 0.10% |
| Non-Traumatic Coma, Brain Compression/Anoxic Damage | 0.20% | 0.00% |
| Personality Disorders | 0.10% | 0.10% |
| Heart Infection/Inflammation, Except Rheumatic | 0.10% | 0.00% |
| Premature Newborns, Including Birthweight 2000-2499 Grams | 0.10% | 0.00% |
| Aspiration and Specified Bacterial Pneumonias and Other Severe Lung Infections | 0.10% | 0.10% |
| Intracranial Hemorrhage | 0.10% | 0.00% |
| Artificial Openings for Feeding or Elimination | 0.10% | 0.00% |
| Respirator Dependence/Tracheostomy Status | 0.00% | 0.00% |
| Completed Pregnancy With Major Complications | 0.20% | 0.10% |
| Chronic Viral Hepatitis C | 0.10% | 0.00% |
| Chronic Pancreatitis | 0.00% | 0.00% |
| Unstable Angina and Other Acute Ischemic Heart Disease | 0.10% | 0.00% |
| Diabetes with Acute Complications | 0.10% | 0.00% |
| Other Premature, Low Birthweight, Malnourished, or Multiple Birth Newborns | 0.10% | 0.00% |
| Bone/Joint/Muscle Infections/Necrosis | 0.10% | 0.10% |
| Lung, Brain, and Other Severe Cancers, Including Pediatric Acute Lymphoid Leukemia | 0.20% | 0.10% |
| Down Syndrome, Fragile X, Other Chromosomal Anomalies, and Congenital Malformation Syndromes | 0.20% | 0.10% |
| Congenital Metabolic Disorders, Not Elsewhere Classified | 0.20% | 0.10% |
| End-Stage Liver Disease | 0.00% | 0.00% |
| Hemiplegia/Hemiparesis | 0.10% | 0.00% |
| Atherosclerosis of the Extremities with Ulceration or Gangrene | 0.00% | 0.00% |
| Acute Liver Failure/Disease, Including Neonatal Hepatitis | 0.00% | 0.00% |
| Monoplegia, Other Paralytic Syndromes | 0.00% | 0.00% |
| End Stage Renal Disease | 0.00% | 0.00% |
| Pervasive Developmental Disorders, Except Autistic Disorder | 0.00% | 0.00% |
| Ectopic and Molar Pregnancy, Except with Renal Failure, Shock, or Embolism | 0.10% | 0.10% |
| Stem Cell, Including Bone Marrow, Transplant Status/Complications | 0.00% | 0.00% |
| Central Nervous System Infections, Except Viral Meningitis | 0.00% | 0.00% |
| Spina Bifida and Other Brain/Spinal/Nervous System Congenital Anomalies | 0.10% | 0.10% |
| Drug Psychosis | 0.00% | 0.00% |
| Quadriplegic Cerebral Palsy | 0.00% | 0.00% |
| Cerebral Palsy, Except Quadriplegic | 0.10% | 0.00% |
| Cerebral Aneurysm and Arteriovenous Malformation | 0.10% | 0.10% |
| Lipidoses and Glycogenosis | 0.00% | 0.00% |
| Parkinson's, Huntington's, and Spinocerebellar Disease, and Other Neurodegenerative Disorders | 0.10% | 0.00% |
| Major Congenital Anomalies of Diaphragm, Abdominal Wall, and Esophagus, Age < 2 | 0.00% | 0.00% |
| Viral or Unspecified Meningitis | 0.00% | 0.00% |
| Reactive and Unspecified Psychosis, Delusional Disorders | 0.10% | 0.10% |
| Premature Newborns, Including Birthweight 1500-1999 Grams | 0.00% | 0.00% |
| Myelodysplastic Syndromes and Myelofibrosis | 0.00% | 0.00% |
| Cystic Fibrosis | 0.00% | 0.00% |
| Pathological Fractures, Except of Vertebrae, Hip, or Humerus | 0.00% | 0.00% |
| Amyotrophic Lateral Sclerosis and Other Anterior Horn Cell Disease | 0.00% | 0.00% |
| Muscular Dystrophy | 0.00% | 0.00% |
| Premature Newborns, Including Birthweight 1000-1499 Grams | 0.00% | 0.00% |
| Aplastic Anemia | 0.00% | 0.00% |
| Extremely Immature Newborns, Including Birthweight 500-749 Grams | 0.00% | 0.00% |
| Osteogenesis Imperfecta and Other Osteodystrophies | 0.00% | 0.00% |
| Intestine Transplant Status/Complications | 0.00% | 0.00% |
| Chronic Kidney Disease, Stage 5 | 0.00% | 0.00% |
| Quadriplegia | 0.00% | 0.00% |
| Prader-Willi, Patau, Edwards, and Autosomal Deletion Syndromes | 0.00% | 0.00% |
| Extremely Immature Newborns, Including Birthweight 750-999 Grams | 0.00% | 0.00% |
| Amputation Status, Lower Limb/Amputation Complications | 0.00% | 0.00% |
| Miscarriage with Complications | 0.00% | 0.00% |
| Lung Transplant Status/Complications | 0.00% | 0.00% |
| Pancreas Transplant Status/Complications | 0.00% | 0.00% |
| Heart Transplant | 0.00% | 0.00% |
| Extremely Immature Newborns, Birthweight < 500 Grams | 0.00% | 0.00% |
| Necrotizing Fasciitis | 0.00% | 0.00% |
| Traumatic Complete Lesion Cervical Spinal Cord | 0.00% | 0.00% |
| Respiratory Arrest | 0.00% | 0.00% |
| Traumatic Complete Lesion Dorsal Spinal Cord | 0.00% | 0.00% |
